# Six-Year Real-World Outcomes of Antivascular Endothelial Growth Factor Monotherapy and Combination Therapy for Various Subtypes of Polypoidal Choroidal Vasculopathy

**DOI:** 10.1155/2019/1609717

**Published:** 2019-12-14

**Authors:** Jingyuan Yang, Mingzhen Yuan, Song Xia, Youxin Chen

**Affiliations:** ^1^Department of Ophthalmology, Peking Union Medical College Hospital, Peking Union Medical College, Chinese Academy of Medical Sciences, Beijing 100730, China; ^2^Department of Ophthalmology, Guizhou Provincial People's Hospital, Guiyang 550002, Guizhou, China

## Abstract

The purpose of this study was to compare 6-year visual outcomes of antivascular endothelial growth factor (anti-VEGF) monotherapy and initial combination therapy of photodynamic therapy (PDT) and anti-VEGF therapy for polypoidal choroidal vasculopathy (PCV) in a Chinese population and to investigate imaging biomarkers associated with visual outcomes. Forty-eight treatment-naive PCV eyes of 46 patients were reviewed retrospectively, which underwent anti-VEGF monotherapy or initial combination therapy. PCV was classified into 2 subtypes. Mean best-corrected visual acuity (BCVA) using logarithm of minimal angle resolution and imaging morphological features was compared. No significant differences of mean BCVA changes were noticed between anti-VEGF monotherapy and combination therapy in either subtype 1 PCV or subtype 2 PCV during 6-year period (all *P* values >0.05). Compared with BCVA at baseline, the mean BCVA at 72 months deteriorated significantly in eyes with subtype 1 PCV (*P* < 0.001), while the mean BCVA at 72 months remained stable in eyes with subtype 2 PCV (*P*=0.941). In subtype 2 PCV eyes with continuous retina pigment epithelium, the mean changes of BCVA in eyes treated with anti-VEGF monotherapy were better than those in eyes treated with combination therapy (*P*=0.020). Anti-VEGF monotherapy and combination therapy for various subtypes of PCV had comparable long-term visual outcomes in most cases in real world. Imaging biomarkers which correlate with visual outcomes and treatment response should be included in the classification of PCV and validated in real world.

## 1. Introduction

Polypoidal choroidal vasculopathy (PCV) is characterized by polypoidal hyperfluorescence with or without a branching vascular network (BVN) in indocyanine green angiography (ICGA), which is the gold standard for diagnosing PCV [1, 2]. Currently, a wide spectrum of treatment options, including antivascular endothelial growth factor (anti-VEGF) therapy, photodynamic therapy (PDT), and various combinations of these therapies, have been performed in real world. Several clinical trials compared various treatment regimens [3–7]. The EVEREST-II study compared the intravitreal injection (IVT) of ranibizumab (IVT-R) and combination of PDT and IVT-R and concluded that combination therapy is preferred to IVR monotherapy [8]. Also, the PLANET study compared IVT aflibercept without and with rescue PDT after 3 months and suggested that no additional benefit was gained in combining with PDT as a rescue therapy [4]. These inconsistent conclusions reveal that more further studies are needed for management of PCV.

Anti-VEGF monotherapy and combination therapy of PDT and anti-VEGF therapy were recommended by recent guidelines and clinical trials [3–5, 9, 10]. Unlike clinical trials which enrolled subjects with restrict criteria prospectively, the efficacy of these treatment regimens needs to be confirmed in real world. Several studies have confirmed the efficacy of anti-VEGF therapies and PDT with additional anti-VEGF therapy for PCV over a long-term period [11–16]. However, long-term outcomes of initial combination therapy of PDT and anti-VEGF therapy in real world have not been reported. And, it has been a consensus that more studies are needed to validate the long-term impact of various classifications of PCV on visual outcomes and treatment regimens [10].

The first aim of the present study was to report the 6-year outcomes of anti-VEGF monotherapy and combination therapy of PDT and anti-VEGF therapy for various subtypes of PCV. The second aim was to investigate imaging biomarkers that might correlate with long-term visual outcomes and treatment response, which should be considered when classifying PCV into various subtypes.

## 2. Materials and Methods

### 2.1. Enrollment of Study Subjects

We retrospectively reviewed 48 eyes of 46 consecutive patients with more than 6 years of follow-up who underwent anti-VEGF monotherapy and combination therapy for PCV at the Department of Ophthalmology, Peking Union Medical College Hospital between May 1, 2010, and May 1, 2013. All patients provided written informed consent after they received an explanation of the treatment. This retrospective study was performed with the approval of the Institutional Review Board of Peking Union Medical College Hospital (reference no. S-K631) and conducted in accordance with the tenets of the Declaration of Helsinki. No identifiable images were used in this retrospective study, and no patient consent was required.

The inclusion criteria were (1) symptomatic macular serosanguinous pigment epithelium detachment (PED) with subfoveal leakage on fluorescein angiography (FA) and (2) presence of polypoidal hyperfluorescence with or without a BVN on ICGA. The exclusion criteria were (1) any previous treatment for PCV, including anti-VEGF therapy, PDT, laser coagulation, or transpupillary thermotherapy; (2) any other concomitant ocular diseases, such as typical neovascular age-related macular degeneration (nvAMD), diabetic retinopathy, retinal artery or vein occlusion, and glaucoma; or (3) retinal pigment epithelium tears or ripping.

### 2.2. Examination

Main outcome measurement was best-corrected visual acuity (BCVA). All patients received a complete ocular examination, including BCVA using logarithm of minimum angle of resolution (logMAR) which was converted from decimal visual acuity measured with tumbling E chart, slit-lamp biomicroscopy, dilated fundus examination, FA and ICGA (Spectralis HRA, Heidelberg Engineering, Heidelberg, Germany), and optical coherence tomography (OCT; 3D-OCT 1000 and 2000, Topcon Corp., Tokyo, Japan, and Spectralis OCT, Heidelberg Engineering, Heidelberg, Germany).

The patients were observed at baseline, every 1 month in the first 3 months, at least every 3 months in the rest of the first year, and at least 6 months in the second to the sixth year. The FA and ICGA were performed at baseline. At every visit, the BCVA, dilated fundus examination, and OCT were performed. The examination data were collected from the baseline and the 1-, 2-, 3-, 6-, 9-, 12-, 18-, 24-, 30-, 36-, 42-, 48-, 54-, 60-, 66-, 72-month (±2 weeks in the last 5 years) follow-ups and were interpreted retrospectively.

PCV was diagnosed with confocal scanning laser ophthalmoscope-based ICGA while subretinal focal polypoidal hyperfluorescence with or without BVN was noticed. PCV was classified into 2 subtypes according to appearances on ICGA and OCT retrospectively: subtype 1 PCV and subtype 2 PCV, which have been found to be correlated with their pathogenesis and visual outcomes [17–19]. The subtype 1 PCV had feeder and draining vessels for polyps, also known as polypoidal CNV or nvAMD related polyps, which did not meet the definition of subtype 2 PCV as described below. Also, the subtype 2 PCV had no apparent feeder or draining vessels, also known as idiopathic PCV or PCV in the narrow sense in previous studies, which presents polypoidal alterations to neovascular or abnormal vascular tissue, usually accompanied by pachychoroid, in the absence of drusen, characteristic pigmentary abnormalities, and geographic atrophy. The greatest linear dimension (GLD) was determined by the ICGA, which included entire polyps and BVNs at the early phase of ICGA, assessed using HRA built-in software. The distance from foveola to the nearest polyp and BVN was also measured. Configuration of polyps was classified into 2 categories according to appearances on ICGA: isolated and interconnected (cluster or string) [20, 21]. The number of polypoidal lesions was also classified into 2 categories: single and multiple. OCT features at baseline included intraretinal fluid, subretinal fluid, and the continuity of external limiting membrane (ELM), ellipsoid zone (EZ), and retina pigment epithelium (RPE). The continuity of the lines or bands corresponding to ELM, EZ, and RPE was detected using dense OCT scans centered on lesions, at least using a 49-line raster scan pattern, 20 × 20 degrees, and multiple scans were performed on each eye. The discontinuity of ELM, EZ, or RPE was defined as a disruption of the corresponding line or band on OCT images. Because ICGA was an invasive examination and was not considered to be performed routinely in clinical practice during follow-up, the recurrence of fluid and exudation was defined as the recurrence of disease activity using OCT [13].

### 2.3. Intervention

Patients who underwent various interventions were enrolled and grouped into 2 groups: anti-VEGF monotherapy and combination therapy. Patients in the anti-VEGF monotherapy group underwent injection of only anti-VEGF agents, including ranibizumab, bevacizumab, and conbercept. After the initial treatment at baseline, repeat treatment of anti-VEGF therapy was applied as needed (pro re nata (PRN)), and conversion of anti-VEGF agents was recorded. Patients in the combination therapy group underwent a session of PDT guided by ICGA and an anti-VEGF injection within 10 days after PDT or on the same day. Retreatment was applied when retinal hemorrhage, intraretinal fluid, or subretinal fluid were observed without treatment. However, for those who presented persistent intra- or subretinal fluid which were resistant to treatment, repeat treatment might be considered not to be performed if patients requested so. The decision was at the physicians' discretion and performed by the physicians in our department. Each treatment was explained detailedly to the patients until patients and we reached an agreement on the treatment plan.

### 2.4. Statistical Analysis

SPSS 25.0 (IBM, Chicago, USA) was used for statistical analysis. Paired *t*-test and 2-sample *t*-test were used for analysis of continuous variables. The chi-squared test was used for categorical variables. Multiple linear regression analysis was performed on related imaging features (continuity of ELM, EZ, and RPE, intraretinal fluid, subretinal fluid, GLD, the distance from foveola to the nearest polyp and BVN, and the number and configuration of polyps), and the changes of BCVA were used as the dependent variable using the stepwise model with the threshold *P* value =0.05 for enter and 0.10 for remove, in which age, gender, and BCVA at baseline were adjusted. Considering that almost all participants had one or several missing data, the missing data were imputed using the last-observation-carried-forward method and compared for consistency with those obtained using observed data. Differences with *P* < 0.05 were considered statistically significant.

## 3. Results

In total, 48 eyes of 46 patients who completed 6-year follow-up visits after the initial treatments were analyzed. The patients' clinical details are listed in Table 1. Age, gender, baseline BCVA, and treatment regimens showed no significant differences between these two subtypes. GLD, distance from foveola to BVN, the distribution of continuous RPE, and configuration and number of polyps showed significant differences between these two subtypes (all *P* values <0.05). Age, gender, baseline BCVA, baseline distance from foveola to BVN and the nearest polyp, the baseline presence of continuous ELM, EZ, and RPE, and intraretinal fluid showed significant differences between various treatment regimens (all *P* values <0.05). The baseline GLD was greater in eyes treated with combination therapy than that in eyes treated with anti-VEGF monotherapy (3554.9 versus 2378.5, *P*=0.018), while baseline subretinal fluid was more common in the eyes treated with anti-VEGF monotherapy than that in the eyes treated with combination therapy significantly (79.2% versus 43.5%, *P*=0.017).

Among the enrolled eyes, 24 eyes received anti-VEGF monotherapy, while the other 23 eyes received combination therapy. The mean number of treatments is summarized in Table 2 according to various subtypes of PCV. The mean number of anti-VEGF therapy showed no significant differences between the treatment regimens of anti-VEGF monotherapy and combination therapy in both subtype 1 PCV and subtype 2 PCV during the follow-up period, except in year 5 in subtype 2 PCV, in which the mean number of anti-VEGF in the regimen of anti-VEGF monotherapy was less than that in the regimen of combination therapy significantly (*P*=0.019). The mean number of anti-VEGF therapy showed no significant differences between subtype 1 PCV and subtype 2 PCV when using the treatment regimens of anti-VEGF monotherapy and combination therapy during the follow-up period, except in year 3 when using the combination therapy (*P*=0.033) and in year 5 when using the anti-VEGF monotherapy (*P*=0.049). Seventeen eyes (36.2%) received conversions between various anti-VEGF agents.

**Figure 1:**
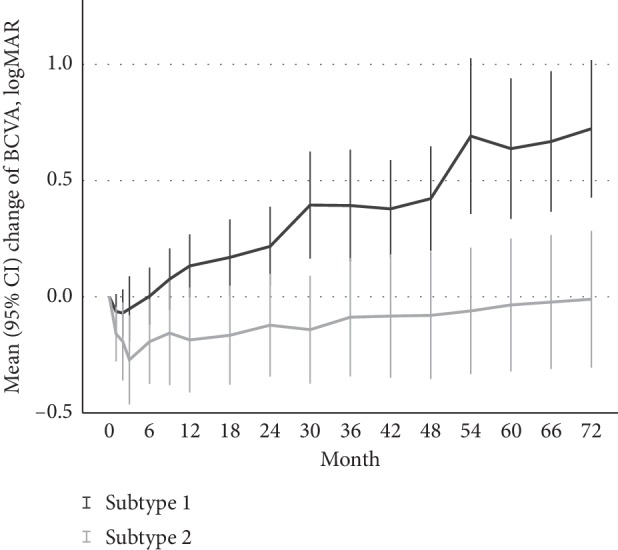
Mean (95% confidence interval) changes of best-corrected visual acuity (BCVA) from baseline using logMAR over 72 months after antivascular endothelial growth factor (VEGF) monotherapy or combination therapy of photodynamic therapy and anti-VEGF therapy for polypoidal choroidal vasculopathy (PCV). The subtype 2 PCV had better mean changes of BCVA than the subtype 1 PCV since month 12 (*P* < 0.05).

Figure 1 shows the mean vision changes over time for both subtype 1 PCV and subtype 2 PCV, and it was found that eyes with subtype 2 PCV had better visual outcomes than eyes with subtype 1 PCV since month 12. The mean BCVA at month 72 deteriorated significantly in eyes with subtype 1 PCV (*P* < 0.001), while the mean BCVA at month 72 remains stable in eyes with subtype 2 PCV (*P*=0.941). However, no significant difference of mean vision change was noticed between various treatment regimens in eyes with either subtype 1 PCV or subtype 2 PCV (all *P* values >0.05) (Supplementary [Supplementary-material supplementary-material-1]).

Recurrence of disease activity was detected in 28 eyes (59.6%). For eyes with subtype 1 PCV, recurrence of disease activity was detected in 6 eyes treated with anti-VEGF monotherapy and 12 eyes treated with combination therapy, and no significant difference was found between various treatment regimens (*P*=0.061). For eyes with subtype 2 PCV, recurrence of disease activity was detected in 4 eyes treated with anti-VEGF monotherapy and 6 eyes treated with combination therapy, and no significant difference was found between various treatment regimens either (*P*=0.222). The number of eyes with intraretinal fluid, subretinal fluid, and macular atrophy at month 72 is shown in Table 3. For subtype 1 PCV, the percentage of macular atrophy in eyes treated with combination therapy was significantly higher than that in eyes treated with anti-VEGF monotherapy (*P*=0.041). The percentage of subretinal fluid at month 72 in eyes treated with anti-VEGF monotherapy was significantly higher than that in eyes treated with combination therapy (*P*=0.046).

Multiple linear regression analysis showed that the mean changes of BCVA during the follow-up period were significantly related to the configuration of polyps (*β* = 0.723; *P* < 0.001) and the continuity of RPE at baseline (*β* = −1.185; *P* < 0.001). In subtype 2 PCV eyes with continuous RPE (examples can be seen in Supplementary [Supplementary-material supplementary-material-1]), the mean changes of BCVA in eyes treated with anti-VEGF monotherapy were better than those in eyes treated with combination therapy (−0.464 versus 0.131; *P*=0.020). However, no significant difference of mean changes of BCVA was found between various treatment regimens in both subtype 1 PCV and subtype 2 PCV in eyes with either isolated or interconnected polyps (all *P* values >0.05).

## 4. Discussion

The current study compared 6-year outcomes of anti-VEGF monotherapy and combination therapy of PDT and anti-VEGF therapy for various subtypes of PCV. The present study showed that eyes with subtype 2 PCV had favorable long-term visual outcomes, and no significant differences of long-term visual outcomes and treatment numbers were found between anti-VEGF monotherapy and initial combination therapy of PDT and anti-VEGF therapy in eyes with either subtype 1 PCV or subtype 2 PCV. Anti-VEGF monotherapy had better visual outcomes than combination therapy for subtype 2 PCV eyes with continuous RPE.

In the current study, anti-VEGF monotherapy and combination therapy had comparable long-term visual outcomes, which was in accordance with the results of the PLANET study [4]. However, combination therapy in real world was considered for eyes with greater severity and activity according to current guidelines [2, 10]. Still, caution should be taken when considering PDT for eyes with PCV because of rare incidences of complications, including subretinal hemorrhage, choroidal infarction, and RPE tear [22–26].

Imaging morphological features that might predict response to therapy and visual outcomes could be regarded as imaging biomarkers in the management of PCV. In this long-term real-world study, we found that anti-VEGF monotherapy for eyes with continuous RPE had better visual outcomes than combination therapy in subtype 2 PCV. Therefore, the continuity or RPE could be taken into consideration when investigating future classification and management of PCV. Neurosensory retina might be affected by abnormal vessels or BVN directly when RPE was discontinuous, and the higher percentage of discontinuous RPE, which refers to alterations of the outer blood-retinal barrier of tight junctions between RPE cells, might contribute to the deteriorated BCVA outcomes [27, 28]. Besides, it has been reported that PDT might lead to choriocapillary occlusion, RPE, and neuroretina injury, which still needs to be validated furtherly using ICGA or OCT angiography [22, 29]. Therefore, we speculated that the dysfunction of RPE and outer layers of neuroretina after PDT might lead to unfavorable visual outcomes. Similarly, PDT might cause choroidal hypoperfusion [30]; therefore, macular atrophy was more common in subtype 1 eyes treated with combination therapy. Additionally, the method of classification for PCV in the current study has aroused attention increasingly in clinical practice [10]. In the present study, the subtype 2 PCV had significantly more favorable long-term visual outcomes when compared with the subtype 1 PCV. Similarly, Jang et al. also reported a better BCVA at baseline and at month 12 after the initial treatment in eyes with subtype 2 PCV than that in eyes with subtype 1 PCV, [17] which accords with our results. Therefore, the current study not only validated the previous classification but also came up with another imaging feature, the continuity of RPE that might correlate with visual outcome and treatment response and could be considered in future classification for PCV. However, it needs to be noted that the classification for PCV is still under investigation because of its complexity. Previous clinicopathological studies have confirmed that both VEGF-positive lesions and VEGF-negative lesions existed in various PCV specimens, [31, 32] which suggested that the pathogenesis of PCV was complicated and eyes with PCV of various imaging morphological features might have different pathogenesis. Therefore, imaging-based classification for PCV needs further investigation and validation, and correlation with visual outcomes and treatment response should be evaluated in detail.

The mean number of anti-VEGF therapies in the present real-world study was less than that in previous studies, which did not classify PCV into various subtypes. An extensive study of the LAPTOP study reported a mean injection number of 14.8 for ranibizumab in the anti-VEGF monotherapy group over 5 years, in which the number of ranibizumab injection was 8.0 in 3 years after the LAPTOP study, while the number of aflibercept was 3.7 [14]. Another Japanese study reported a mean injection number of 18.2 for ranibizumab over 6 years [16]. Although conversive therapy of anti-VEGF agents might help reduce the injection number, other influential factors in real world should be taken into consideration. Firstly, the retreatment criteria in real world were not entirely the same as those of previous studies, and the retreatment decisions were made on the eyes with only explicit signs of recurrence rather than the eyes with potential signs or only decreased BCVA. In the current study, retreatment might not be performed on eyes with persistent intraretinal fluid or subretinal fluid which were resistant to treatment if patients requested so in real world. And, in the present retrospective study without a strict prospective protocol, eyes of less activity and severity were allowed to visit us every 6 months in real world. Secondly, the enrolled Chinese patients might bear the financial burden, and the anti-VEGF agents used in this study have not been paid by the national medical insurance during majority of the follow-up period, which indeed reduced the patients' therapeutic compliance. Therefore, the mean number of anti-VEGF injections in real world was less than previous studies, especially in developing countries.

Our study has several limitations, including the relatively small patient number. Since long-term follow-up is difficult in ordinary clinical practice, the number of patients in each treatment group among various subtypes of PCV is relatively small, so that our results might need to be confirmed by further studies which include more subjects. Although we have examined the distribution of various treatment regimens among different subtypes of PCV, bias due to the small number of subjects seems to be unavoidable in such a study, which is similar to previous studies of long-term treatment for PCV [11–16]. Moreover, because of the retrospective nature of this study, long-term randomized clinical trials and prospective real-world studies are needed to investigate more effective treatment regimens for PCV according to imaging features. Besides, because the correlation between polypoidal choroidal vasculopathy and pachychoroid spectrum diseases was noticed after the time point that the enrolled eyes received treatment, data about choroidal morphology were not collected using OCT. Fortunately, a great number of studies about choroidal morphology in eyes with PCV have been published. Additionally, early treatment diabetic retinopathy study charts were not used for visual examination in this study because in clinical practice tumbling E charts were commonly used in China. However, logMAR was used to measure the changes of BCVA for statistical analysis in this study, which has been well accepted universally. Moreover, the OCT scan pattern we used might miss some subtle ELM, EZ, or RPE disruptions, so that the differences of visual outcomes between PCV eyes with and without continuous RPE might be slightly less significant. Because the imaging technique has developed during these years, more imaging biomarkers associated with visual outcomes need further investigation using current devices. Furthermore, only Chinese patients were enrolled, and worldwide multicenter investigations might be needed to study PCV in real world.

## 5. Conclusion

In conclusion, the 6-year outcomes of anti-VEGF monotherapy and initial combination therapy for PCV were reviewed. Our study demonstrated that both treatment regimens showed comparable visual outcomes over 72-month follow-up, except that anti-VEGF monotherapy had more favorable visual outcomes for subtype 2 PCV eyes with continuous RPE. Because this study was a retrospective review with limited size, large, long-term, and prospective randomized studies are needed to investigate the optimal management for PCV. Also, imaging-based classification for PCV which correlates with visual outcomes and treatment response needs further investigation and validation.

## Figures and Tables

**Table 1:** Baseline characteristics of the participants with various subtypes of polypoidal choroidal vasculopathy.

	Subtype 1	Subtype 2	*P*
Patients (*n*)	24	23	
Gender (*n*), female/male	8/16	10/13	0.556
Age (year), mean ± SD	64.3 ± 7.6	61.3 ± 8.0	0.184
Best-corrected visual acuity (logMAR), mean ± SD	0.54 ± 0.36	0.55 ± 0.37	0.965
Optical coherence tomography features			
Continuous external limiting membrane (*n*)	2	3	0.666
Continuous ellipsoid zone (*n*)	0	2	0.234
Continuous retinal pigment epithelium (*n*)	6	17	0.001
Intraretinal fluid (*n*)	15	11	0.375
Subretinal fluid (*n*)	11	18	0.036
Indocyanine green angiography features			
Greatest linear dimension (*μ*m), mean ± SD	3859.7 ± 1625.6	1862.0 ± 1011.0	<0.001
The distance from foveola to the nearest polyp (*μ*m), mean ± SD	1897.2 ± 1126.4	655.5 ± 540.7	<0.001
The distance from foveola to branching vascular network (*μ*m), mean ± SD	255.1 ± 497.4	340.7 ± 433.2	0.551
Configuration of polyps (*n*), isolated/interconnected	7/17	18/5	0.001
Number of polyps (*n*), single/multiple	1/23	11/12	0.001
Treatment regimen (*n*), anti-VEGF monotherapy/combination therapy	11/13	13/10	0.564

logMAR, logarithm of minimum angle of resolution; SD, standard deviation; VEGF, vascular endothelial growth factor.

**Table 2:** Mean (standard deviation) number of anti-VEGF therapy and PDT in the regimen of anti-VEGF monotherapy and the regimen of combination therapy for various subtypes of polypoidal choroidal vasculopathy during the 6-year period.

	Subtype 1			Subtype 2		
	Anti-VEGF monotherapy	Combination therapy		Anti-VEGF monotherapy	Combination therapy	
		Anti-VEGF therapy	PDT		Anti-VEGF therapy	PDT
Year 1	2.73 (1.10)	4.62 (3.10)	1.00 (0)	2.62 (2.57)	2.40 (1.84)	1.00 (0)
Year 2	1.09 (1.14)	1.77 (1.88)	0 (0)	0.62 (1.19)	1.10 (1.10)	0.20 (0.42)
Year 3	1.82 (1.94)	1.54 (1.76)	0.15 (0.38)	0.69 (1.55)	0.40 (0.70)	0.10 (0.32)
Year 4	1.27 (2.00)	1.46 (1.56)	0 (0)	0.23 (0.60)	1.60 (2.91)	0.20 (0.42)
Year 5	0.91 (1.22)	1.00 (1.08)	0 (0)	0 (0)	0.90 (0.99)	0 (0)
Year 6	0.91 (1.38)	0.62 (0.51)	0 (0)	0.15 (0.38)	0.70 (1.06)	0 (0)
Total	8.73 (6.17)	11.00 (4.24)	1.15 (0.38)	4.31 (3.71)	7.10 (5.97)	1.50 (0.71)

PDT, photodynamic therapy; VEGF, vascular endothelial growth factor.

**Table 3:** Anatomical outcomes for 72 months in eyes with PCV by optical coherence tomography.

	Subtype 1		Subtype 2	
	Anti-VEGF monotherapy	Combination therapy	Anti-VEGF monotherapy	Combination therapy
Intraretinal fluid	5	6	3	1
Subretinal fluid	1	4	6	0
Macular atrophy	0	5	3	4

PCV, polypoidal choroidal vasculopathy; VEGF, vascular endothelial growth factor.

## Data Availability

The data used to support the findings of this study are available from the corresponding author upon request.

## Supplementary Materials

Supplementary Materials Supplementary Figure 1: mean changes of best-corrected visual acuity over 72 months for two subtypes of PCV after treatment. Supplementary Figure 2: examples of continuity of retinal pigment epithelium.

## References

[1] Yannuzzi L. A., Sorenson J., Spaide R. F., Lipson B. (1990). Idiopathic polypoidal choroidal vasculopathy (IPCV). *Retina*.

[2] Koh A. H. C., Chen L.-J., Chen S.-J. (2013). Polypoidal choroidal vasculopathy. *Retina*.

[3] Oishi A., Kojima H., Mandai M. (2013). Comparison of the effect of ranibizumab and verteporfin for polypoidal choroidal vasculopathy: 12-month LAPTOP study results. *American Journal of Ophthalmology*.

[4] Wong T. Y., Ogura Y., Lee W. K. (2019). Efficacy and safety of intravitreal Aflibercept for polypoidal choroidal vasculopathy: two-year results of the Aflibercept in polypoidal choroidal vasculopathy study. *American Journal of Ophthalmology*.

[5] Takahashi K., Ohji M., Terasaki H. (2018). Efficacy and safety of ranibizumab monotherapy versus ranibizumab in combination with verteporfin photodynamic therapy in patients with polypoidal choroidal vasculopathy: 12-month outcomes in the Japanese cohort of EVEREST II study. *Clinical Ophthalmology*.

[6] Koh A., Lee W. K., Chen L.-J. (2012). Everest study. *Retina*.

[7] Gomi F., Oshima Y., Mori R. (2015). Initial versus delayed photodynamic therapy in combination with ranibizumab for treatment of polypoidal choroidal vasculopathy. *Retina*.

[8] Koh A., Lai T. Y. Y., Takahashi K. (2017). Efficacy and safety of ranibizumab with or without verteporfin photodynamic therapy for polypoidal choroidal vasculopathy. *JAMA Ophthalmology*.

[9] Qian T., Li X., Zhao M., Xu X. (2018). Polypoidal choroidal vasculopathy treatment options: a meta-analysis. *European Journal of Clinical Investigation*.

[10] Cheung C. M. G., Lai T. Y. Y., Ruamviboonsuk P. (2018). Polypoidal choroidal vasculopathy. *Ophthalmology*.

[11] Saito M., Iida T., Kano M., Itagaki K. (2014). Five-year results of photodynamic therapy with and without supplementary antivascular endothelial growth factor treatment for polypoidal choroidal vasculopathy. *Graefe’s Archive for Clinical and Experimental Ophthalmology*.

[12] Nishikawa K., Oishi A., Hata M. (2019). Four-year outcome of Aflibercept for neovascular age-related macular degeneration and polypoidal choroidal vasculopathy. *Scientific Reports*.

[13] Chang Y. S., Kim J. H., Kim K. M. (2016). Long-term outcomes of anti-vascular endothelial growth factor therapy for polypoidal choroidal vasculopathy. *Journal of Ocular Pharmacology and Therapeutics*.

[14] Miyamoto N., Mandai M., Oishi A. (2018). Long-term results of photodynamic therapy or ranibizumab for polypoidal choroidal vasculopathy in LAPTOP study. *British Journal of Ophthalmology*.

[15] Kang H. M., Koh H. J. (2013). Long-term visual outcome and prognostic factors after intravitreal ranibizumab injections for polypoidal choroidal vasculopathy. *American Journal of Ophthalmology*.

[16] Hikichi T. (2018). Six-year outcomes of antivascular endothelial growth factor monotherapy for polypoidal choroidal vasculopathy. *British Journal of Ophthalmology*.

[17] Jang J. W., Kim J. M., Kang S. W., Kim S. J., Bae K., Kim K. T. (2019). Typical polypoidal choroidal vasculopathy and polypoidal choroidal neovascularization. *Retina*.

[18] Kawamura A., Yuzawa M., Mori R., Haruyama M., Tanaka K. (2013). Indocyanine green angiographic and optical coherence tomographic findings support classification of polypoidal choroidal vasculopathy into two types. *Acta Ophthalmologica*.

[19] Coscas G., Lupidi M., Coscas F. (2015). Toward a specific classification of polypoidal choroidal vasculopathy: idiopathic disease or subtype of age-related macular degeneration. *Investigative Opthalmology & Visual Science*.

[20] Cackett P., Wong D., Yeo I. (2009). A classification system for polypoidal choroidal vasculopathy. *Retina*.

[21] Hou J., Tao Y., Li X.-x., Zhao M.-w. (2011). Clinical characteristics of polypoidal choroidal vasculopathy in Chinese patients. *Graefe’s Archive for Clinical and Experimental Ophthalmology*.

[22] Lo Giudice G., De Belvis V., Piermarocchi S., Galan A., Prosdocimo G. (2008). Acute visual loss and chorioretinal infarction after photodynamic therapy combined with intravitreal triamcinolone. *European Journal of Ophthalmology*.

[23] Akaza E., Yuzawa M., Matsumoto Y., Kashiwakura S., Fujita K., Mori R. (2007). Role of photodynamic therapy in polypoidal choroidal vasculopathy. *Japanese Journal of Ophthalmology*.

[24] Chan W.-M., Lam D. S. C., Lai T. Y. Y. (2004). Photodynamic therapy with verteporfin for symptomatic polypoidal choroidal vasculopathy. *Ophthalmology*.

[25] Kim S.-W., Oh J., Oh I. K., Huh K. (2009). Retinal pigment epithelial tear after half fluence PDT for serous pigment epithelial detachment in central serous chorioretinopathy. *Ophthalmic Surgery, Lasers, and Imaging*.

[26] Klais C. M., Ober M. D., Freund K. B. (2005). Choroidal infarction following photodynamic therapy with verteporfin. *Archives of Ophthalmology*.

[27] Chung M., Lee S., Lee B. J., Son K., Jeon N. L., Kim J. H. (2018). Wet-AMD on a chip: modeling outer blood-retinal barrier in vitro. *Advanced Healthcare Materials*.

[28] Cunha-Vaz J., Bernardes R., Lobo C. (2011). Blood-retinal barrier. *European Journal of Ophthalmology*.

[29] Husain D., Kramer M., Kenny A. G. (1999). Effects of photodynamic therapy using verteporfin on experimental choroidal neovascularization and normal retina and choroid up to 7 weeks after treatment. *Investigative Ophthalmology & Visual Science*.

[30] Schmidt-Erfurth U. M., Michels S. (2003). Changes in confocal indocyanine green angiography through two years after photodynamic therapy with verteporfin. *Ophthalmology*.

[31] Nakajima M., Yuzawa M., Shimada H., Mori R. (2004). Correlation between indocyanine green angiographic findings and histopathology of polypoidal choroidal vasculopathy. *Japanese Journal of Ophthalmology*.

[32] Nakashizuka H., Mitsumata M., Okisaka S. (2008). Clinicopathologic findings in polypoidal choroidal vasculopathy. *Investigative Opthalmology & Visual Science*.

