# Supplementary material for: Six-Year Real-World Outcomes of Antivascular Endothelial Growth Factor Monotherapy and Combination Therapy for Various Subtypes of Polypoidal Choroidal Vasculopathy
Source: J Ophthalmol. 2019 Dec 14;2019:1609717. doi: 10.1155/2019/1609717 (PMC6948291; doi:10.1155/2019/1609717)
Supplement: Supplementary Materials — Supplementary Figure 1: mean changes of best-corrected visual acuity over 72 months for two subtypes of PCV after treatment. Supplementary Figure 2: examples of continuity of retinal pigment epithelium. [file 1609717.f1.docx]

### Supplementary Materials

**
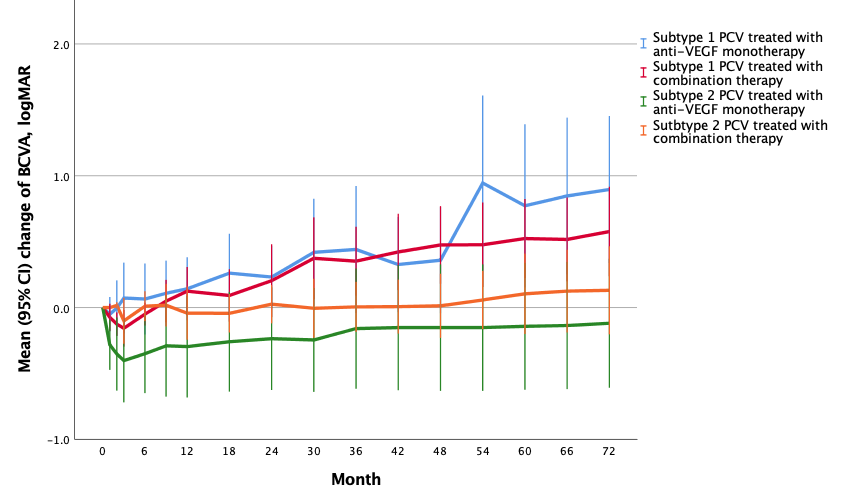
**

**Supplementary Figure 1.** Mean (95% confidence interval) changes of best-corrected visual acuity (BCVA) from baseline using logMAR over 72 months after anti-vascular endothelial growth factor (VEGF) monotherapy or combination therapy of photodynamic therapy and anti-VEGF therapy for two subtypes of polypoidal choroidal vasculopathy (PCV). No significant difference of mean vision change was noticed between various treatment regimens in eyes with either subtype 1 PCV or subtype 2 PCV at each visit (all *P*-values > 0.05).

**
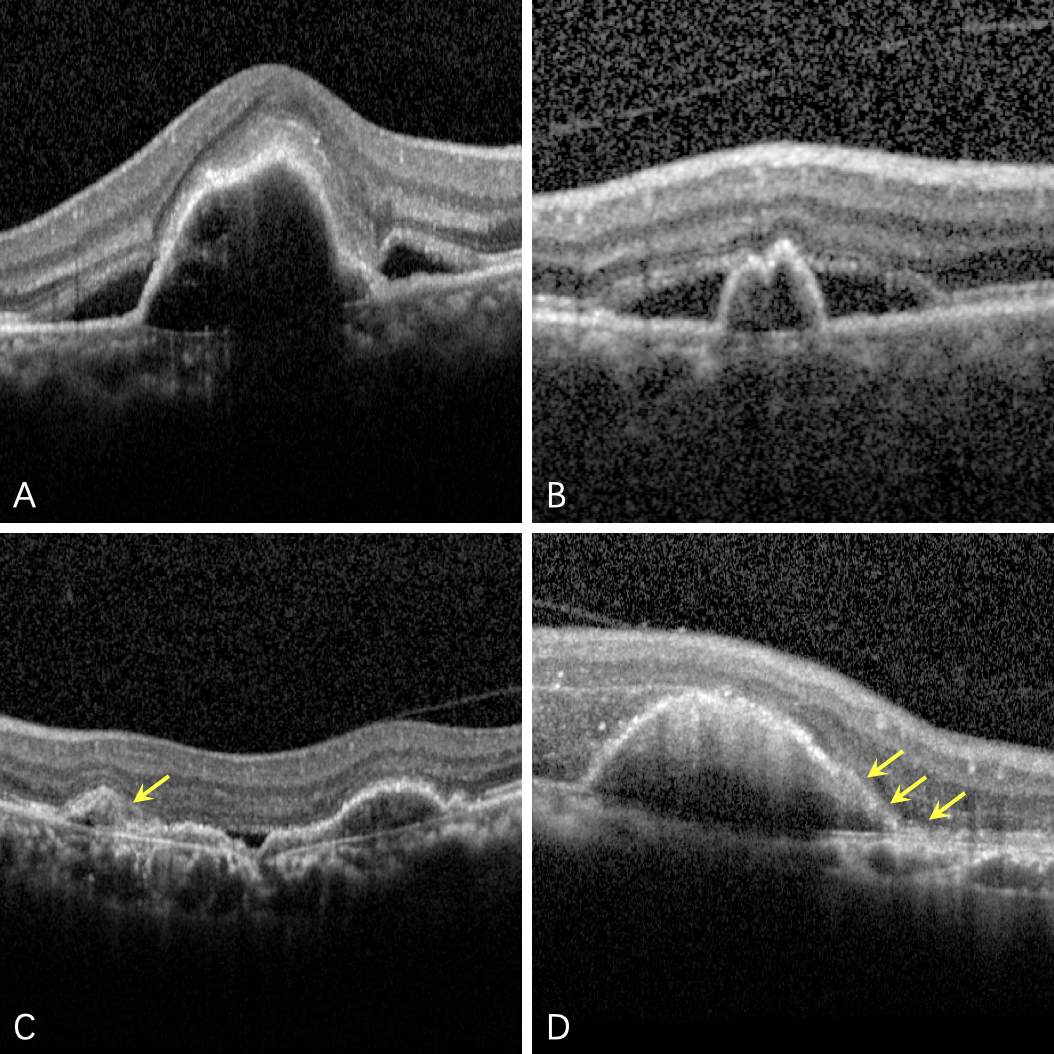
**

**Supplementary Figure 2.** Examples of continuous and discontinuous of retinal pigment epithelium (RPE) in eyes with polypoidal choroidal vasculopathy (PCV). Continuous RPE is shown in (A) and (B). Although the line of RPE in (A) is rough, no obvious break of RPE could be detected. The integrity of the line of RPE in (B) is well preserved. Discontinuous RPE is shown in (C) and (D). Fibrovascular PED is shown in (C), and a break of RPE is noticed (yellow arrow), which locates upon the abnormal vessels. In (D), multiple breaks of RPE are seen (yellow arrow). The line of RPE is rough, which is similar to the line of RPE in (A), but discontinuity can be detected at RPE.
